# Alp7/TACC recruits kinesin-8–PP1 to the Ndc80 kinetochore protein for timely mitotic progression and chromosome movement

**DOI:** 10.1242/jcs.160036

**Published:** 2015-01-15

**Authors:** Ngang Heok Tang, Takashi Toda

**Affiliations:** Laboratory of Cell Regulation, Cancer Research UK, London Research Institute, Lincoln's Inn Fields Laboratories, 44 Lincoln’s Inn Fields, London WC2A 3PX, UK

**Keywords:** Alp7/TACC, Kinesin-8, Protein phosphatase I, Ndc80, Anaphase A, Spindle assembly checkpoint/fission yeast

## Abstract

Upon establishment of proper kinetochore–microtubule attachment, the spindle assembly checkpoint (SAC) must be silenced to allow onset of anaphase, which is when sister chromatids segregate equally to two daughter cells. However, how proper kinetochore–microtubule attachment leads to timely anaphase onset remains elusive. Furthermore, the molecular mechanisms of chromosome movement during anaphase A remain unclear. In this study, we show that the fission yeast Alp7/TACC protein recruits a protein complex consisting of the kinesin-8 (Klp5–Klp6) and protein phosphatase 1 (PP1) to the kinetochore upon kinetochore–microtubule attachment. Accumulation of this complex at the kinetochore, on the one hand, facilitates SAC inactivation through PP1, and, on the other hand, accelerates polewards chromosome movement driven by the Klp5–Klp6 motor. We identified an *alp7* mutant that had specific defects in binding to the Klp5–Klp6–PP1 complex but with normal localisation to the microtubule and kinetochore. Consistent with our proposition, this mutant shows delayed anaphase onset and decelerated chromosome movement during anaphase A. We propose that the recruitment of kinesin-8–PP1 to the kinetochore through Alp7/TACC interaction plays a crucial role in regulation of timely mitotic progression and chromosome movement during anaphase A.

## INTRODUCTION

During mitosis, the microtubules grow from the opposite spindle poles and bind to the kinetochore, a multi-protein structure found on the centromeric DNA (Cheeseman and Desai, 2008; Takeuchi and Fukagawa, 2012). The kinetochore–microtubule attachment is monitored by a surveillance mechanism known as the spindle assembly checkpoint (SAC) (Musacchio and Salmon, 2007; Lara-Gonzalez et al., 2012). When errors in kinetochore–microtubule attachment are detected, the SAC, in which Mad2 represents a central protein, is activated to allow time for correction of faulty attachment through inhibiting the anaphase-promoting complex/cyclosome (APC/C) ubiquitin ligase (He et al., 1997; Hwang et al., 1998; Kim et al., 1998). Subsequently, upon establishment of proper kinetochore–microtubule attachment, the SAC must be silenced (or deactivated) to allow anaphase onset and segregation of sister chromatids into two daughter cells (Lesage et al., 2011). The type I protein phosphatase (PP1) has been shown to play a crucial role in checkpoint silencing (Pinsky et al., 2009; Vanoosthuyse and Hardwick, 2009). However, how PP1 imposes SAC silencing coincident with the establishment of end-on attachment remains unexplored.

In the past decades, numerous studies have revealed the importance of the outer kinetochore complex component Ndc80/Hec1 in regulating microtubule attachment. The N-terminal region of Ndc80 interacts directly with the spindle microtubules (Wei et al., 2007; Ciferri et al., 2008). In addition, the Ndc80 internal loop helps to recruit different regulatory proteins to ensure accurate chromosome segregation (Nilsson, 2012; Tang and Toda, 2013; Tang and Toda, 2014). In human cells, the licensing factor Cdt1 binds to the Ndc80 loop to alter the Ndc80 conformation to an extended form (Varma et al., 2012), whereas localisation of the spindle and kinetochore-associated (Ska) complex to the kinetochore is important for the establishment of end-on attachment (Zhang et al., 2012). In budding yeast, the Dam1 complex localises to the kinetochore through the Ndc80 internal loop and plays crucial roles in the conversion of lateral to end-on attachment (Maure et al., 2011). In fission yeast, the Ndc80 internal loop recruits both the Dis1/TOG and the Alp7/TACC–Alp14/TOG complex (Hsu and Toda, 2011; Tang et al., 2013).

Members of the conserved XMAP215/ch-TOG (colonic hepatic tumour overexpressed gene) microtubule-associated protein family (Garcia et al., 2001; Nakaseko et al., 2001; Ohkura et al., 2001; Kinoshita et al., 2002) are known to be plus-end tracking proteins (+TIPs) with microtubule polymerase activities (Brouhard et al., 2008; Al-Bassam and Chang, 2011; Widlund et al., 2011; Al-Bassam et al., 2012; Podolski et al., 2014). Both Dis1 and Alp14 belong to this family and share essential functions (Nabeshima et al., 1995; Garcia et al., 2001; Nakaseko et al., 2001; Garcia et al., 2002a). Despite this, there are several differences between Dis1 and Alp14. First, Alp14, but not Dis1, forms a complex with Alp7 protein (Sato et al., 2004). Second, in early mitosis, Dis1 localises to the kinetochore (Nakaseko et al., 2001; Hsu and Toda, 2011), whereas Alp14 is initially targeted to the spindle pole body (SPB) through Alp7, then localises to spindle microtubules and finally reaches the kinetochores upon attachment (Sato et al., 2004; Sato and Toda, 2007; Kakui et al., 2013; Okada et al., 2014; Tang et al., 2013; Tang et al., 2014; Zheng et al., 2014).

We have previously shown that the temperature-sensitive *ndc80-21* mutant, which has an L405P mutation within the loop, is defective in recruiting Dis1 to unattached kinetochores (Hsu and Toda, 2011), whereas another loop mutant *ndc80-NH12*, which has an F420S mutation, is deficient in localising the Alp7–Alp14 complex to the kinetochore upon attachment (Tang et al., 2013). Failure to recruit Dis1 to the kinetochore causes spindle collapse and prolonged activation of the SAC in the *ndc80-21* mutant, whereas reduced levels of the Alp7–Alp14 complex at the kinetochores in the *ndc80-NH12* mutant induces one of two phenotypes, either mitotic delay (type I) or chromosome mis-segregation with marginal delay (type II). These studies have provided further understanding of an important event in early mitosis, namely how microtubules are attached to the kinetochore. Despite these studies, two key questions in the later parts of mitosis remain unresolved. Firstly, how the SAC is silenced in response to proper kinetochore-microtubule attachment, and secondly, which molecules are responsible for driving chromosome movement during anaphase A.

In this study, we identified the new roles of Alp7 in regulating anaphase onset and chromosome movement. We showed that Alp7 recruits a protein complex consisting of the plus-end-directed kinesin-8 motors Klp5–Klp6 and PP1 to the kinetochores, which in turn promote chromosome movement and SAC silencing, respectively. We propose that Alp7 acts as a central hub for crucial protein–protein interactions at the kinetochores. Our study provides a new insight into the molecular organisation at the kinetochore–microtubule interface and its importance in proper chromosome segregation.

## RESULTS

### PP1^Dis2^ deletion corrects microtubule-kinetochore mal-attachment and suppresses temperature sensitivity of the *ndc80-NH12* mutant

**Fig. 1.**
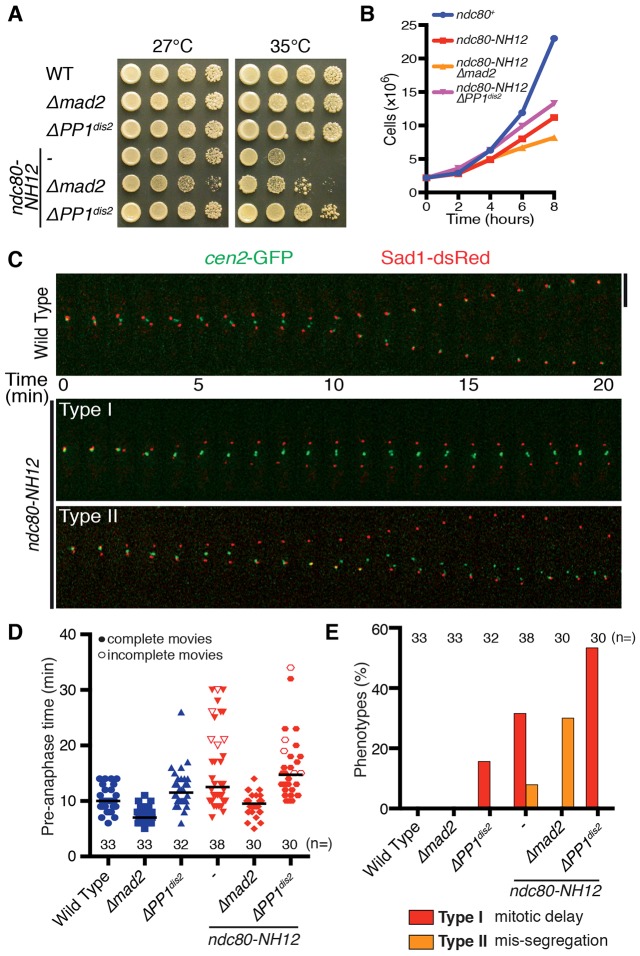
**Depletion of PP1^Dis2^ suppresses *ndc80-NH12* growth defects.** (A) Suppression of *ndc80-NH12* by *PP1^dis2^*, but not *mad2*, deletion. Serial dilution spot tests were performed on rich agar medium and incubated at the temperatures indicated for 3 days. (B) Rescue in liquid culture. Strains were grown in rich medium at 27°C and shifted to 36°C. (C) Time-lapse imaging of centromere 2 behaviour. Mitotic cells containing *cen2*–GFP and Sad1–dsRed (SPB) were imaged at 36°C. Two distinct phenotypes were identified (Type I: mitotic delay; Type II: *cen2* mis-segregation). Representative images from the *ndc80-NH12* mutant are shown. Scale bar: 5 µm. See supplementary material Movie 1 (wild type), Movie 2 (Type I) and Movie 3 (Type II). (D) Time spent in pre-anaphase at 36°C. Pre-anaphase time was defined as the duration spent from pole separation until the completion of anaphase A (when sister chromatids separate and attach to the opposite poles). The horizontal lines depict the median. Note that the values of pre-anaphase time in *ndc80-NH12* or *ndc80-NH12 PP1^dis2^* are underestimated, as we could not see anaphase onset in several samples in these mutants by the end of filming (shown as unfilled triangles or circles respectively). (E) Quantification of phenotypes observed. Cells that spent ≥15 min in pre-anaphase were classified as having mitotic delay (Type I).

The temperature sensitive *ndc80-NH12* mutant displays massive chromosome mis-segregation that can be ascribed to the reduced levels of Alp7–Alp14 at the kinetochore (Tang et al., 2013). We first addressed whether the abrogation of the checkpoint activation or silencing had any impact on the *ndc80-NH12* mutant. Intriguingly, deletion of PP1^Dis2^ (the kinetochore-localised isoform of PP1; Dis2 is one of the two PP1 catalytic subunits in fission yeast) but not Mad2, rescued the growth defects in *ndc80-NH12* (Fig. 1A,B). Consistent with the role of PP1^Dis2^ in SAC silencing (Vanoosthuyse and Hardwick, 2009), ∼15% of *ΔPP1^d^^is2^* cells displayed mitotic delay (Fig. 1C–E; Type I) in the otherwise wild-type background. A further delay in mitotic progression was induced in the *ndc80-NH12 ΔPP1^dis2^* double mutant cells compared to that of each *ndc80-NH12* or *ΔPP1^dis2^* single mutant (Fig. 1C–E, and see supplementary material Movies 1–3). In line with the suppression of growth defects, no chromosome mis-segregation was observed in the *ndc80-NH12 ΔPP1^dis2^* double mutants, whereas ∼30% of *ndc80-NH12 Δmad2* double mutant cells showed centromere 2 mis-segregation (Fig. 1D,E; Type II). These results suggest that deletion of PP1^Dis2^ in *ndc80-NH12* mutant cells suppresses its phenotypes by imposing further mitotic delay, to allow extra time for correction of erroneous kinetochore–microtubule attachment. In sharp contrast, deletion of PP1^Dis2^ was synthetically lethal with *ndc80-21*, whereas Mad2 deletion had no impact on its temperature sensitivity; however, it resulted in massive loss of cell viability (supplementary material Fig. S1A; Hsu and Toda, 2011). Therefore, PP1^Dis2^ deletion is specifically beneficial for *ndc80-NH12*.

Fission yeast contains two homologues of PP1, known as Dis2 and Sds21, in which Dis2 plays a major role at the centromeric regions (Ohkura et al., 1989; Alvarez-Tabarés et al., 2007). Consistently, *ndc80-NH12* phenotypes were suppressed only weakly by *ΔPP1^sds21^* (supplementary material Fig. S1B). Apart from PP1^Dis2^, Bub3 has also been shown to facilitate SAC silencing in fission yeast (Tange and Niwa, 2008; Vanoosthuyse and Hardwick, 2009; Windecker et al., 2009). In fact, *Δbub3* rescued temperature sensitivity of the *ndc80-NH12* mutant, although less effectively than *ΔPP1^dis2^* (supplementary material Fig. S1C). These results indicate that prolonged SAC-dependent mitotic delay, especially by the PP1^Dis2^ deletion, ameliorates lethal chromosome segregation errors in the *ndc80-NH12* mutant.

### The Klp5–Klp6–PP1 complex localises to the kinetochore through Ndc80–Alp7

We next addressed the precise mechanism by which PP1 molecules spatially recognise the Ndc80–Alp7–Alp14-mediated kinetochore–microtubule attachment. PP1 has been shown to bind to the kinetochore protein Spc7 (also known as KNL1, Spc105 and Blinkin) and the heterodimeric kinesin-8 motor Klp5–Klp6 (Liu et al., 2010; Meadows et al., 2011; Rosenberg et al., 2011). Klp5–Klp6 motors localise to the mitotic kinetochore (Garcia et al., 2002b; West et al., 2002). We therefore envision that PP1 plays crucial roles in checkpoint silencing, specifically at the kinetochores. Given that the deletion of the PP1-binding motif in Spc7 is lethal (Meadows et al., 2011), we mutated the PP1-binding domains of Klp5 and Klp6 with their motor domains intact [Klp5(PP1^mut^) and Klp6(PP1^mut^), respectively; supplementary material Fig. S2A].

**Fig. 2.**
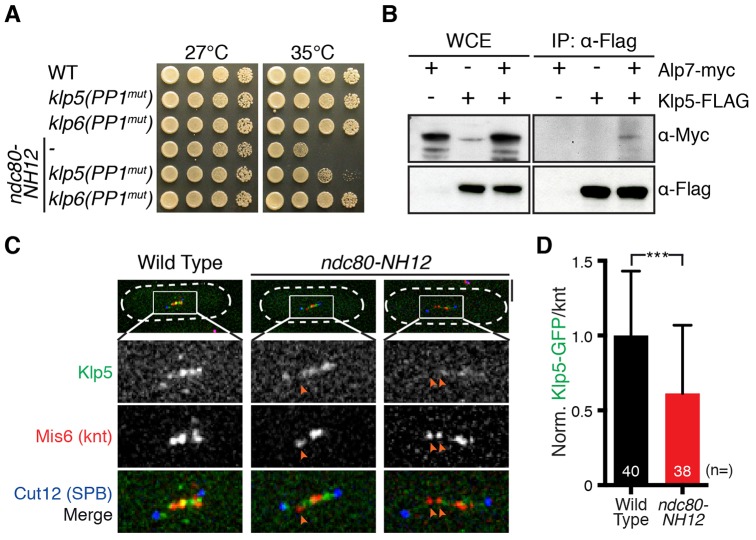
**Alp7 interacts with Klp5 to localise the Klp5–Klp6 complex to the kinetochore.** (A) Disruption of PP1-binding sites in Klp5 or Klp6 suppresses *ndc80-NH12*. Serial dilution spot tests were performed on rich agar medium and incubated at the temperatures indicated for 3 days. (B) Co-immunoprecipitation (IP) between Alp7 and Klp5. Protein extracts (4 mg) were prepared from metaphase-arrested *cut9-665* cells containing Alp7–Myc and/or Klp5–3FLAG. WCE, whole-cell extract (15 µg). (C) Klp5 delocalises from the kinetochores in the *ndc80-NH12* mutant. Wild-type and *ndc80-NH12* mutant cells were incubated at 36°C for 90 min and fixed with methanol. Cell shapes are outlined with discontinuous white lines. Orange arrowheads mark delocalisation of Klp5–GFP from the kinetochore. Scale bar: 5 µm. (D) Quantification of GFP signals at the kinetochores. Intensities of Klp5–GFP on each kinetochore (using the Mis6–2mRFP signal as a reference) were quantified. Klp5–GFP intensities for wild-type samples were set as 1, and those of *ndc80-NH12* samples were calculated accordingly. Sample numbers (*n*) are indicated. Results are mean±s.d. ***P<0.001 (two-tailed Student's *t*-test).

Similar to *ΔPP1^dis2^*, disruption of the PP1-binding sites within Klp5 or Klp6 also alleviated *ndc80-NH12* growth defects, although Klp6(PP1^mut^) suppressed the defects better than Klp5(PP1^mut^) (Fig. 2A). It is of note that Klp5 contains two PP1-binding consensus sequences, whereas Klp6 contains only one (Meadows et al., 2011), suggesting that the binding affinity of Klp5 and Klp6 to PP1 might not be the same. We envision that this altered property leads to the different efficacy of suppression towards *ndc80-NH12*. It is worth pointing out that the temperature sensitivity of *ndc80-NH12* is not ameliorated by the full deletion of Klp5 (or Klp6), nor the truncation of the Klp5–Klp6 motor domains (supplementary material Fig. S2B). This is consistent with the notion that Klp5–Klp6 motors play crucial roles in regulating spindle length and microtubule dynamics in addition to PP1 recruitment (Garcia et al., 2002a; West et al., 2002; Grissom et al., 2009; Syrovatkina et al., 2013). Overall, our data suggest that Klp5–Klp6 delivers PP1 to the attached kinetochores, thereby promoting SAC silencing.

Next, we sought to determine how Klp5–Klp6 plays a role at the kinetochore. As the Ndc80 loop interacts with Alp7, which has multiple protein–protein interaction modules (Tang et al., 2013; Thakur et al., 2013), we posited that Klp5–Klp6 might localise to the kinetochores through Alp7. Accordingly, we examined the interaction between Klp5 and Alp7 *in vivo*. Intriguingly, we found that Alp7–Myc co-immunoprecipitated with Klp5–FLAG during mitosis (Fig. 2B). In agreement with this, a significant reduction of Klp5–Klp6 localisation to the kinetochore was observed in the *ndc80-NH12* mutant (Fig. 2C,D), which compromises the recruitment of Alp7–Alp14 to the kinetochore (Tang et al., 2013). It is intriguing that, on the one hand, *ndc80-NH12* cells display reduced levels of Alp7–Klp5–Klp6–PP1 at the kinetochore and that, on the other hand, this mutant is suppressed by loss of PP1 at the kinetochore (through PP1^Dis2^ deletion or *klp5* and/or *klp6* mutants defective in PP1 binding). We speculate that the residual amount of PP1^Dis2^ in *ndc80-NH12* is sufficient to attenuate SAC activation, leading to chromosome mis-segregation. In any case, the results shown here imply that Klp5–Klp6 localises to the kinetochores through interaction with the Alp7–Alp14 complex.

### The *alp7-LA6* mutant ameliorates the temperature sensitivity of the *ndc80-NH12* mutant

**Fig. 3.**
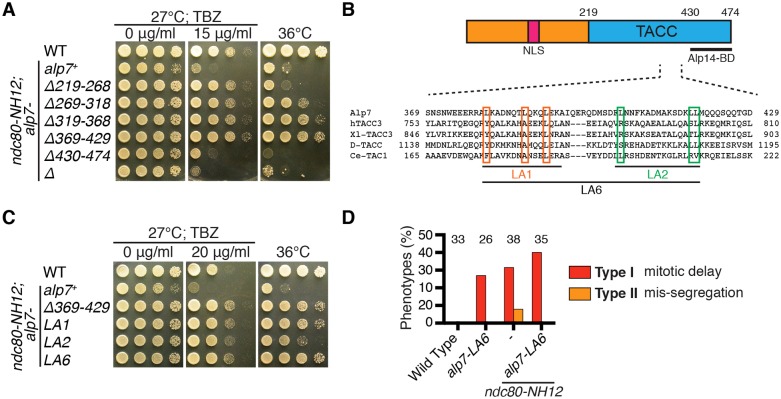
**Isolation and characterisation of the *alp7-LA6* mutant.** (A) Alp7 truncation analysis. The TACC domain of Alp7 (amino acids 219–474) were systematically truncated and transformed into *ndc80-NH12* cells. Note that *alp7-Δ430–474* mimics *Δalp7*, as the amino acid 430–474 region is essential for binding to Alp14 (Sato et al., 2009). (B) Amino acid sequence alignments of TACC family members from fission yeast, human, frog, fly and worm are shown. Position of mutations in LA1, LA2 and LA6 mutants (leucine to alanine replacements) are indicated with boxes. The extreme C-terminal 45 amino acid sequences of Alp7 are necessary and sufficient for Alp14 binding (Sato et al., 2004), whereas the region between amino acids 300 and 360 is important for targeting to the SPB (Tang et al., 2014; Zheng et al., 2014). No *alp7* mutants, either point or truncation, have thus far been identified that are specifically defective in kinetochore localisation. The kinetochore-localising region (shown as knt?) could overlap with the SPB-targeting sequence. (C) Suppression of *ndc80-NH12* by *alp7* mutants. *alp7* mutants carrying different mutations were created in the *ndc80-NH12* background. (D) Quantification of phenotypes. The percentage of cells showing phenotypes as described in Fig. 1C was quantified. Note that samples for wild-type and *ndc80-NH12* are the same as in Fig. 1E. Sample numbers (*n*) are indicated.

We then sought to find the Klp5–Klp6-binding region within Alp7. We contemplated that the Alp7 mutant protein with defective Klp5 binding but intact Ndc80-loop-binding activity would suppress the *ndc80-NH12* phenotypes due to the loss of PP1 at the kinetochores. We performed systematic truncation analysis of the Alp7 TACC domain in the *ndc80-NH12* mutant background. This analysis led to the identification of two *alp7* mutants (*alp7-Δ319-368* and *alp7-Δ369-429*) that rescued the *ndc80-NH12* mutant growth defects (Fig. 3A). We have recently shown that Alp7-Δ319-368 fails to localise to the SPB and kinetochores during mitosis, resulting in SAC-dependent prolonged mitotic progression (Tang et al., 2014). Rescue of *ndc80-NH12* by *alp7-Δ319–368* further supported the idea that SAC-mediated mitotic delay ameliorates lethal chromosome mis-segregation defects in this mutant.

Intriguingly, the *alp7-Δ369-429* mutant showed modest, but reproducible resistance to the microtubule drug thiabendazole (TBZ) (Fig. 3A; supplementary material Fig. S3A); hyper-resistance to TBZ is a characteristic of the *Δklp5/6* mutant (either each single mutant or the double deletion mutant) (West et al., 2001; Garcia et al., 2002b). We hypothesised that the amino acid region 369–429 is important for Klp5–Klp6 binding. The C-terminal TACC domain (amino acids 219–474) consists of multiple coiled-coil motifs, representing protein–protein interaction sites, in which the hydrophobic residues present in α-helixes are crucial (Burkhard et al., 2001). Therefore, we replaced several leucine residues with alanine residues in the region of 369–429 (Fig. 3B). We found that the three mutants, *alp7-LA1*, *alp7-LA2* and *alp7-LA6*, all suppressed *ndc80-NH12* phenotypes to various degrees (Fig. 3C). All three mutants, similar to the *alp7-Δ369-429* mutant, also showed increased resistance to TBZ (Fig. 3C; supplementary material Fig. S3B).

Given that *alp7-LA6* suppressed *ndc80-NH12* phenotypes the most, we decided to hereafter use this mutant for further analysis. Visual inspection of centromere 2 movement in the *ndc80-NH12 alp7-LA6* double mutant showed no *cen2* mis-segregation, but instead displayed longer mitotic delay than either *ndc80-NH12* or *alp7-LA6* single mutant (Fig. 1C; Fig. 3D). This profile is similar, if not identical, to that shown in the *ndc80-NH12 ΔPP1^dis2^* double mutant (see Fig. 1C–E). These results suggest that the introduction of *alp7-LA6* into *ndc80-NH12* mutant cells induces further mitotic delay, potentially through reduction of Klp5–Klp6–PP1 at the kinetochores. In stark contrast, introduction of *alp7-LA6* did not rescue *ndc80-21* (supplementary material Fig. S3C).

### The *alp7-LA6* mutant fails to bind to Klp5–Klp6

**Fig. 4.**
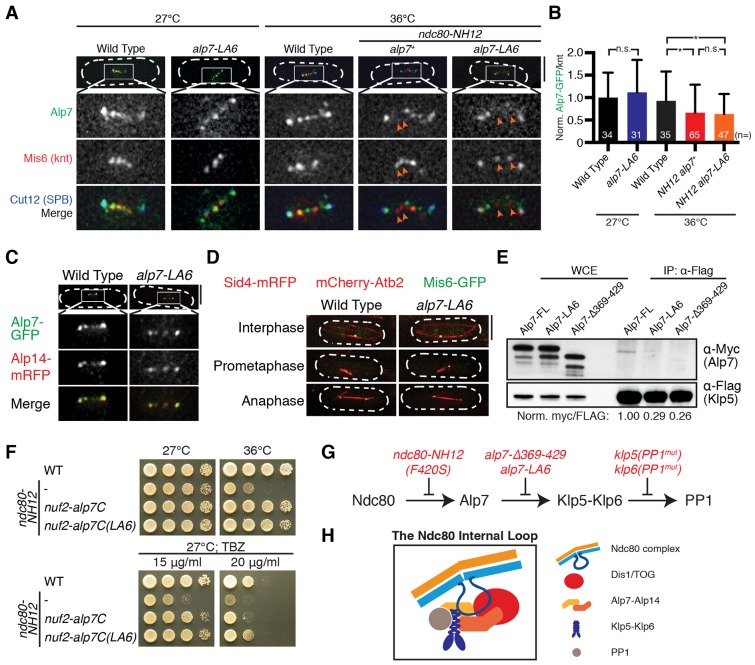
**Alp7-LA6 shows reduced binding to Klp5, but retains normal kinetochore, SPB and Alp14 binding.** (A) Alp7-LA6 localises normally to the kinetochores. Arrows show delocalisation of Alp7 from the kinetochores. Note that Alp7 localises to not only kinetochores (marked by Mis6) but also to SPBs (marked by Cut12). (B) Quantification of Alp7–GFP (wild-type and LA6) signals at the kinetochores. Alp7–GFP intensities for wild-type samples were set as 1, and those of other samples were calculated accordingly. Sample numbers (*n*) are shown. Results are mean±s.d. *P<0.05, n.s., not significant (two-tailed Student's *t*-test). (C) Visualisation of Alp7–Alp14 localisation. Wild-type and *alp7-LA6* cells were grown at 27°C and fixed with methanol before imaging. (D) Microtubule morphology of wild-type and *alp7-LA6* cells. Cells were grown at 27°C and visualised. Scale bars: 5 µm. (E) Co-immunoprecipitation (IP) between Klp5 and wild-type or mutant Alp7. Immunoprecipitation was performed as in Fig. 2B, using 8 mg of cell extracts. Relative intensities (shown below the blot) of each Alp7–Myc band were quantified using the corresponding Klp5–3Flag band as a control. WCE, whole-cell extract. (F) Tethering of the C-terminal TACC domain of Alp7-LA6 to the kinetochores leads to TBZ resistance. The C-terminus of Alp7 or Alp7-LA6 was fused to Nuf2 at the native locus. Note that *ndc80-NH12 nuf2-alp7C(LA6)* is more resistant to TBZ than *ndc80-NH12 nuf2-alp7C*. (G) A summary of Ndc80–Alp7–Klp5–Klp6–PP1 interactions and the defects in individual mutants. (H) The Ndc80 internal loop binds to Dis1/TOG and Alp7–Alp14. Binding of Alp7 to Klp5 brings Klp5–Klp6 motor proteins close to the Ndc80 internal loop. Klp5–Klp6 binding to PP1 plays important roles in SAC silencing.

During mitosis, Alp7 localises to the SPB, kinetochores and microtubules in a manner that is dependent on its TACC domain and interaction with Alp14 (Sato et al., 2004; Ling et al., 2009; Sato et al., 2009; Tang et al., 2013). We then examined colocalisation of Alp7-LA6 with the SPB, kinetochores and Alp14. No significant differences in localisation to the kinetochore were observed in the *alp7-LA6* mutant compared to wild-type cells (Fig. 4A,B). Furthermore, in the *ndc80-NH12* background, the levels of Alp7-LA6 were reduced to the same extent as wild-type Alp7 (Fig. 4A,B). In addition, the *alp7-LA6* mutant showed precise colocalisation with the SPB and Alp14, and did not display obvious spindle microtubule abnormalities (Fig. 4C,D). These observations confirm that Alp7-LA6 retains normal functions at the SPB and kinetochores. Thus, we concluded that the amino acids 369–429 in Alp7 are largely responsible for the binding to the Klp5–Klp6–PP1 complex. To further scrutinise this, we performed another co-immunoprecipitation between Klp5 and Alp7, Alp7-LA6 or Alp7-Δ369–429. As expected, binding of Alp7-LA6 and Alp7-Δ369-429 to Klp5 was reduced significantly (by >70%) compared to Alp7 (Fig. 4E). Taken together, our results indicate that the *alp7-LA6* mutant is specifically defective in Klp5 binding, but retains normal binding to Alp14, the SPB and kinetochores.

### Alp7 connects Ndc80 to the kinesin-8–PP1 complex

We reasoned that the binding of Alp7 and Klp5 plays important roles in SAC silencing and controlling chromosome movement specifically at the kinetochores. To test this, we targeted the C-terminal TACC domain (amino acids 219–474, Alp7C) of Alp7 or Alp7-LA6 to the kinetochore component Nuf2, by fusing the *alp7C* gene to the 3′ end of the *nuf2^+^* gene under the control of the endogenous *nuf2^+^* promoter. As reported previously, forced targeting of Alp7C to the kinetochores suppressed *ndc80-NH12* phenotypes, in a manner mediated by recruitment of Alp14 (Tang et al., 2013). Intriguingly, targeting of Alp7C(LA6) to the kinetochores not only suppressed the temperature sensitivity of the *ndc80-NH12* mutant, but also conferred TBZ resistance to the mutant cells, similar to the *alp7-LA6* single mutant and reminiscent of the *Δklp5/6* mutant (Fig. 4F). It is of note that targeting of Alp7C(LA6) to the kinetochore did not result in obvious TBZ resistance when wild-type Ndc80 was present in the cell [in *ndc80^+^ nuf2-alp7C(LA6)*]. We propose that the Ndc80 internal loop is a platform to load different microtubule-associated proteins (MAPs) (Fig. 4G,H). The localisation of different MAPs to the Ndc80 internal loop region brings them close to the plus end of microtubules, thereby regulating microtubule–kinetochore attachment (Nilsson, 2012; Tang and Toda, 2013)

### Recruitment of Klp5–Klp6–PP1 to the kinetochores promotes rapid chromosome movement during anaphase A

**Fig. 5.**
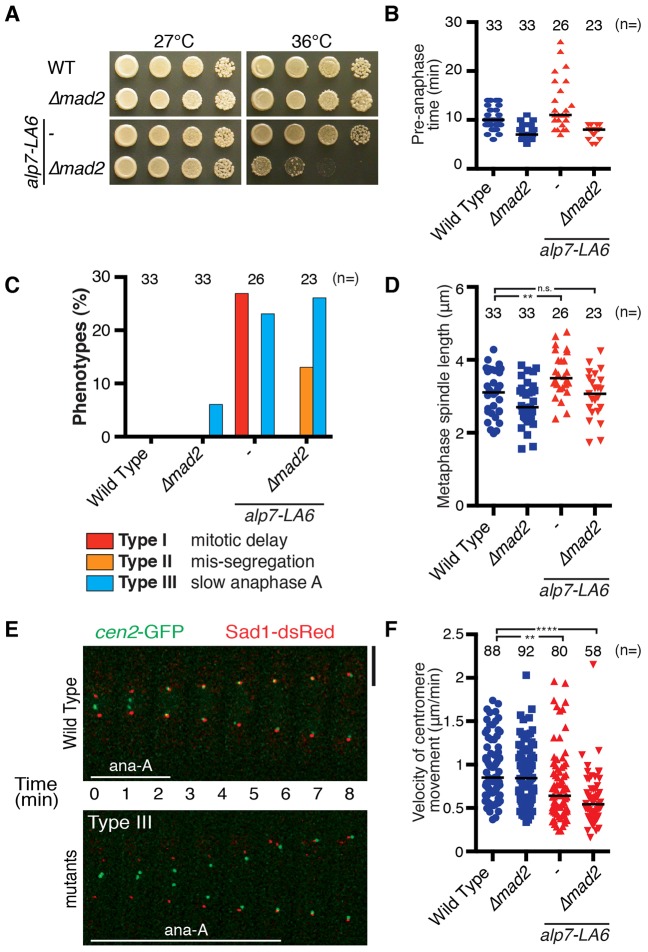
**The *alp7-LA6* mutant shows delayed mitotic progression and slow chromosome movement during anaphase A.** (A) Deletion of Mad2 in the *alp7-LA6* mutant displayed temperature sensitivity. (B) Time indicated strains are in pre-anaphase at 36°C. (C) Quantification of phenotypes. Note that samples for wild type and *alp7-LA6* are the same as in Fig. 1E and Fig. 3D, respectively. Cells spending ≥3 min in anaphase A were classified as Type III (refer to Fig. 5E). (D) Metaphase spindle length at 36°C. (E) Visualisation of *cen2* movement. Representative images of anaphase duration in wild type and *alp7-LA6* cells are shown. Scale bar, 5 µm. Refer to supplementary material Fig. S4 for complete mitotic progression. See also supplementary material Movie S4. (F) Velocity of centromere movement during anaphase A. Sample numbers (*n*) are shown. The horizontal lines in B,D and F depict the median. **P<0.01; ****P<0.0001; n.s., not significant (two-tailed Student's *t*-test).

Having seen delayed anaphase onset in the *alp7-LA6* mutant (Fig. 3D), we then tested whether deletion of Mad2 had any effect on the *alp7-LA6* mutant. Intriguingly, the *alp7-LA6 Δmad2* double mutants showed growth defects at 36°C (Fig. 5A). The absence of Mad2 abolished the mitotic delay observed in the *alp7-LA6* mutant (Fig. 5B), and ∼10% of the double mutant cells displayed chromosome mis-segregation (Fig. 5C; Type II). Furthermore, metaphase spindle length was significantly increased in the *alp7-LA6* mutant compared to wild-type cells (Fig. 5D), in line with the notion that Klp5–Klp6 motors are refractory regulators of mitotic spindle length (Garcia et al., 2002a; West et al., 2002; Syrovatkina et al., 2013). This phenotype was also reversed by deletion of Mad2 (Fig. 5D). These results indicate that the SAC-dependent mitotic delay observed in the *alp7-LA6* mutant is essential to correct faulty kinetochore–microtubule attachment.

By careful inspection of mitotic progression profiles of the *alp7-LA6* mutant, we found that polewards chromosome movement during anaphase A is markedly delayed in the mutant cells (Fig. 5C,E; supplementary material Fig. S4; supplementary material Movie 4; Type III). Detailed analysis of centromere movement revealed that velocities of *cen2* movement during anaphase A were reduced in *alp7-LA6* and *alp7-LA6 Δmad2* cells, indicating that delayed anaphase A is independent of the SAC (Fig. 5F). Remarkably, *alp7-LA6* cells underwent anaphase B (elongation of pole-to-pole microtubules) prior to the completion of anaphase A (supplementary material Fig. S4). These observations imply that a reduced level of Klp5–Klp6 at the kinetochores slows down chromosome movement during anaphase A (when microtubules depolymerise after SAC silencing), consistent with a previous study showing the ability of these motor proteins to transport cargo along depolymerising microtubules *in vitro* (Grissom et al., 2009).

## DISCUSSION

**Fig. 6.**
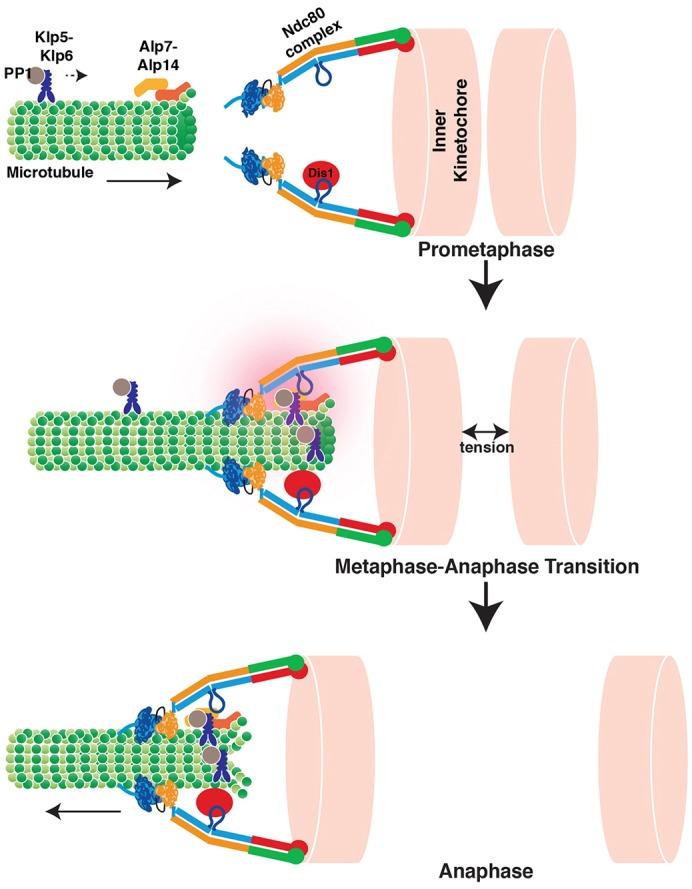
**The Ndc80 internal loop binds to Dis1 and Alp7–Alp14, thereby recruiting the Klp5–Klp6 complex to control mitotic progression.** A schematic view of mitotic progression from prometaphase to anaphase A. In prophase, Dis1 localises to the Ndc80 internal loop. Microtubules nucleate, assemble and elongate from the SPB (not shown) towards the kinetochores. The Alp7–Alp14 complex is delivered by the microtubules. In metaphase, this complex reaches the Ndc80 internal loop, thereby providing a robust kinetochore-microtubule attachment. Alp7 loads the Klp5–Klp6–PP1 complex to the attached kinetochore. Recruitment of the Klp5–Klp6–PP1 complex facilitates silencing of the spindle assembly checkpoint, thereby inducing timely anaphase onset. The Klp5–Klp6 complex then couples chromosome movement to depolymerising microtubule plus-ends to ensure efficient anaphase chromosome movement.

Despite the identification of the essential players required for SAC silencing (e.g. PP1), how proper kinetochore–microtubule attachment leads to SAC silencing and anaphase onset remains a long-standing question. Our work indicates that Alp7 plays a central role in this process. We show that the localisation of Alp7 to the kinetochores through Ndc80 leads to the dual recruitment of Alp14 and the kinesin-8 Klp5–Klp6–PP1 complex. Based upon the current and previous results, we propose the following molecular pathway that leads to faithful chromosome segregation upon kinetochore–microtubule attachment (depicted in Fig. 6). In early mitosis, the Ndc80 internal loop interacts with Dis1 to stabilise microtubules and safeguard the initial attachment of the kinetochore to spindle microtubules. This interaction then allows Alp7–Alp14 to localise to the kinetochores, leading to the establishment of amphitelic kinetochore–microtubule attachment. Following this, binding of the Klp5–Klp6–PP1 complex to Alp7 results in accumulation of the complex at the attached kinetochore. PP1 at the kinetochore–microtubule interface will then stimulate anaphase onset, by silencing the SAC. In addition, clustered kinesin-8 motors couple chromosome movement to depolymerising microtubule plus-ends, thereby driving timely chromosome movement towards the spindle poles in anaphase A.

Previous studies have shown that PP1^Dis2^ plays a crucial role in counteracting the Aurora B kinase and silencing the SAC (Vanoosthuyse and Hardwick, 2009; Meadows et al., 2011). However, these studies were conducted in the β-tubulin-defective *nda3-KM311* background (Hiraoka et al., 1984), in which the spindle microtubules are absent from the cell. Here, we showed that PP1^Dis2^ is also important for SAC silencing under unperturbed conditions; deletion of PP1^Dis2^ causes mitotic delay in the otherwise wild-type fission yeast cells (Fig. 1D,E). We further showed that this SAC-dependent delay is beneficial for cell survival and growth in the *ndc80-NH12* mutant, by ameliorating the chromosome mis-segregation phenotype. To our knowledge, this is the first example showing that induced SAC-dependent mitotic delay by PP1 deletion rescues the chromosome segregation defects of a mutant.

Klp5 and Klp6 are known to localise to the mitotic kinetochore (Garcia et al., 2002b; West et al., 2002), but the precise mechanism and physiological significance for this localisation remained to be explored. Mitotic roles for Klp5 and Klp6 are proposed mainly from the defective phenotypes of complete deletion mutants, including the microtubule dynamics and chromosome alignment in metaphase prior to anaphase A (Garcia et al., 2002a; West et al., 2002; Sanchez-Perez et al., 2005). In this study, we have shown the importance of the Ndc80–Alp7 connection for Klp5–Klp6 localisation to the kinetochores. Furthermore, by identifying the *alp7-LA6* mutant that is specifically defective in the recruitment of Klp5–Klp6 to the kinetochores, we have uncovered the role of Klp5–Klp6 at the kinetochores in promoting rapid polewards chromosome movement during anaphase A.

Previous work in yeast, worm and human cells has highlighted the roles of PP1 in SAC silencing by binding to Spc7/Spc105/KNL1 (Liu et al., 2010; Meadows et al., 2011; Rosenberg et al., 2011; Espeut et al., 2012). However, although the Knl1 family members possess *in vitro* microtubule-binding activities (Cheeseman et al., 2006), these activities do not appear directly involved in the establishment of kinetochore–microtubule attachment per se (Espeut et al., 2012), whereas that of Ndc80 does (Guimaraes et al., 2008; Miller et al., 2008; Alushin et al., 2012; Nilsson, 2012; Tang and Toda, 2013). Furthermore, there is solid evidence for the existence of another PP1-targeting molecule(s) besides Spc105 (Rosenberg et al., 2011), and indeed Klp5–Klp6-bound PP1 regulated SAC silencing (Meadows et al., 2011). Our study, therefore, fills the gap by linking these two events, the establishment of proper kinetochore–microtubule attachment and concurrent SAC silencing that are performed by recruitment of Klp5–Klp6–PP1 to the Ndc80-bound Alp7 connection. Indeed, recent studies have shown that PP1 does bind the human kinesin-8 Kif18A and, furthermore, that disruption of its PP1-binding site results in a delay in anaphase onset, suggesting the conserved role of kinesin-8–PP1 in SAC silencing (De Wever et al., 2014; Häfner et al., 2014). It would be intriguing to see whether the conserved TACC members take part in SAC signalling and, furthermore, whether kinesin-8s regulate chromosome movement during anaphase A in mammalian systems.

Although the budding yeast counterpart to Klp5–Klp6, Kip3, is a microtubule depolymerase (Gupta et al., 2006; Varga et al., 2006), it is still unclear whether Klp5–Klp6 possesses microtubule-depolymerising activity (Grissom et al., 2009; Erent et al., 2012). Nevertheless, it is firmly established that these molecules promote microtubule dynamicity *in vivo* (Unsworth et al., 2008). It would be interesting to test whether Klp5–Klp6 have the ability to bind to the Alp7-Ndc80 complex *in vitro* and deliver this complex along depolymerising microtubules.

Misregulation (both overproduction and downregulation) of Ndc80/Hec1, TACC, TOG and kinesin-8s protein levels are associated with the grade of tumour in different cancers (Diaz-Rodríguez et al., 2008; Wu et al., 2008; Rath and Kozielski, 2012; Ha et al., 2013). Mis-regulation of these proteins leads to chromosome mis-segregation and aneuploidy, a hallmark of cancer (Tang and Toda, 2014). However, the molecular basis of how these proteins are linked to cancer formation remains largely elusive. Here, we show a plausible link for how Ndc80, TACC-TOG and kinesin-8 proteins work in concert at the kinetochore. Our study provides a new insight into the kinetochore–microtubule interface and has implications for future therapeutics and drug development.

## MATERIALS AND METHODS

### Yeast genetics, strains and general methodology

The strains used in this study are listed in supplementary material Table S1. Standard methods for yeast genetics and molecular biology were used (Moreno et al., 1991; Bähler et al., 1998).

### Constructions of various *alp7* mutants

A strain with green fluorescence protein (GFP) and G418-resistant marker (*kan^r^*) integrated into the C terminus of Alp7 was constructed (Sato et al., 2004). The *alp7^+^-GFP-kan^r^* fragment purified from this strain was used as a template to make different *alp7* mutants used in this study. Systematic truncation of the TACC domain (amino acids 219–474) of Alp7 was carried out. DNA fragments encoding Alp7-Δ219-268-GFP-kan^r^, Alp7-Δ269-318-GFP-kan^r^, Alp7-Δ319-368-GFP-kan^r^, and Alp7-Δ369-429-GFP-kan^r^ were amplified by two-step PCR. Strain with Alp7-Δ430-474-GFP-kan^r^ (Alp7-ΔC45-GFP-kan^r^) was as previously constructed (Sato et al., 2009). These DNA fragments were transformed into wild-type cells (strain 513) to replace the endogenous gene expressed from the original locus of *alp7^+^*. Different *alp7* mutants (*alp7-LA1*, *alp7-LA2* and *alp7-LA6*) were constructed using site-directed two-step PCR.

### Isolation of *klp5(PP1^mut^)* and *klp6(PP1^mut^)* mutants

A strain with Klp5-3FLAG-kan^r^ was constructed by integrating the *klp5-3FLAG-kan^r^* DNA fragment into the original locus of *klp5^+^*. The *klp5-3FLAG-kan^r^* fragment was purified from this strain and used as a template for construction of the *klp5(PP1^mut^)* strain. Site-directed mutagenesis was performed as described above, by referring to the mutation sites previously reported (Meadows et al., 2011). The same process was repeated to make the *klp6(PP1^mut^)* strain, using *klp6-GFP-kan^r^* DNA fragment as a template (Unsworth et al., 2008).

### Preparation of synchronous cell culture

For microscopy purpose (Fig 1C; Fig. 2C, Fig. 3A; Fig. 4A), cells were synchronised using hydroxyurea (HU)-induced S-phase arrest and release. Cell cultures were treated with 12.5 mM HU at 25°C for 4 hours, filtered and incubated in HU-free rich medium at 36°C for 1–2 hours. For immunoprecipitation assays (Fig 2B; Fig. 4E), cells were synchronised in mitosis using a *cut9-665* strain (Yamashita et al., 1996). A *cut9-665* mutant that contains Klp5–3Flag with Alp7–Myc, Alp7-LA6–Myc or Alp7-Δ369-429–Myc was used. Cells were grown in rich medium at 25°C and shifted to 36°C for 2.5 hours.

### Immunoprecipitation assay

Immunoprecipitation was performed as previously described (Tang et al., 2013). Yeast cells were broken with glass beads at 4°C in a FastPrep FP120 apparatus (setting 5.5, 25 s, 2×). The protein extracts were collected after 15 minutes of centrifugation at 13,000 ***g*** at 4°C. The indicated amount of protein extracts were then added to protein A Dynabeads coated with anti-Flag antibodies (F7425, Sigma-Aldrich). For immunoblotting, anti-Flag (M2, Sigma-Aldrich) and anti-Myc (MMS150R, Covance) antibodies were used.

### Microscopy

Microscopy was performed as previously described (Tang et al., 2013). Live-cell imaging was done on lectin-coated, glass-bottomed microwell dishes supplemented with rich medium. Fixed-cell imaging was done on glass slides with coverslips. Images taken using the Olympus IX70 wide-field inverted epifluorescence microscope were deconvolved and compressed into a two-dimensional (2D) projection using the DeltaVision-softWoRx maximum-intensity algorithm. Images were taken as 14 sections along the *z*-axis at 0.3-µm intervals.

### Quantification of fluorescence signal intensity

Fluorescence signals were quantified using maximum intensity after images were merged into single projection using DeltaVision-softWoRx as described above. Background signals in the vicinity of the fluorescence spot were subtracted.

### Statistical analysis

Results are presented as mean±s.d. (sample numbers are shown in each figure). *P<0.05; **P<0.01, ***P<0.001, ****P<0.0001, n.s., not significant (two-tailed unpaired Student's *t*-test).

## Supplementary Material

Supplementary Material
