# Supplementary material for: Alp7/TACC recruits kinesin-8–PP1 to the Ndc80 kinetochore protein for timely mitotic progression and chromosome movement
Source: J Cell Sci. 2015 Jan 15;128(2):354–63. doi: 10.1242/jcs.160036 (PMC4294777; doi:10.1242/jcs.160036)
Supplement: Supplementary Material [file supp_128_2_354__index.html]

Alp7/TACC recruits kinesin-8–PP1 to the Ndc80 kinetochore protein for timely mitotic progression and chromosome movement — Supplementary Material 

# Alp7/TACC recruits kinesin-8–PP1 to the Ndc80 kinetochore protein for timely mitotic progression and chromosome movement

## JCS160036 Supplementary Material

**Files in this Data Supplement:**

- **Supplementary Material**
