# Supplementary material for: Alp7/TACC recruits kinesin-8–PP1 to the Ndc80 kinetochore protein for timely mitotic progression and chromosome movement
Source: J Cell Sci. 2015 Jan 15;128(2):354–63. doi: 10.1242/jcs.160036 (PMC4294777; doi:10.1242/jcs.160036)
Supplement: Supplementary Material [file supp_128.2.354_JCS160036.pdf]

Supplementary Materials

Alp7/TACC recruits kinesin-8-PP1 to the Ndc80 kinetochore protein for timely mitotic progression and chromosome movement

Ngang Heok Tang & Takashi Toda

Laboratory of Cell Regulation, Cancer Research UK, London Research Institute, Lincoln's Inn Fields Laboratories, 44 Lincoln's Inn Fields, London WC2A 3PX, UK.

Table S1. Fission yeast strains used in this study

Strains	Genotypes	Figures used
513	h^{-} leu1 ura4	1A-B, 2A, 4F, 5A, S1A-C, S2B, S3B
NHT139	h^{+} mad2::ura4^{+} leu1 ura4 his2	1A, 5A, S1A
NHT1042	h^{-} dis2::ura4^{+} leu1 ura4	1A, S1B-C
NHT080	h^{-} ndc80-NH12-kan^{r} leu1 ura4	1A, 1B, 2A, 4F, S1A-C, S2B, S3C
NHT400	h^{-} ndc80-NH12-kan^{r} mad2::ura4^{+} leu1 ura4	1A-B, S1A
NHT1072	h^{-} ndc80-NH12-kan^{r} dis2::ura4^{+} leu1 ura4	1A-B, S1B-C
NHT942	h^{-} alp7-myc-kan^{r} cen2::hph^{r}-lacOp his7^{+}::lacI-GFP-ura4^{+} sad1^{+}-dsRed-leu2^{+} leu1 ura4	1C-E, 3D, 5B-F, S4
NHT1201	h^{-} ndc80-NH12-kan^{r} alp7-myc-hph^{r} cen2::hph^{r}-lacOp his7^{+}::lacI-GFP-ura4^{+} sad1^{+}-dsRed-leu2^{+} leu1 ura4	1C-E, 3D
NHT497	h^{-} mad2::ura4^{+} cen2::hph^{r}-lacOp his7^{+}::lacI-GFP-ura4^{+} sad1^{+}-dsRed-leu2^{+} leu1 ura4	1C-E , 5B-F, S3
NHT1403	h^{-} dis2::ura4^{+} cen2::hph^{r}-lacOp his7^{+}::lacI-GFP-ura4^{+} sad1^{+}-dsRed-leu2^{+} leu1 ura4	1C-E
NHT522	h^{-} ndc80-NH12-kan^{r} mad2::ura4^{+} cen2::hph^{r}-lacOp his7^{+}::lacI-GFP-ura4^{+} sad1^{+}-dsRed-leu2^{+} leu1 ura4	1C-E
NHT1318	h^{-} ndc80-NH12-kan^{r} dis2::ura4^{+} cen2::hph^{r}-lacOp his7^{+}::lacI-GFP-ura4^{+} sad1^{+}-dsRed-leu2^{+} leu1 ura4	1C-E
NHT1151	h^{-} klp5(PP1^{mut})-FLAG-kan^{r} leu1 ura4	2A

NHT1123	*h*$^-$ *klp6(PP1*^{mut}*)-GFP-kan*^{r} *leu1 ura4*	2A
NHT1215	*h*$^-$ *klp5(PP1*^{mut}*)-FLAG-kan*^{r} *ndc80-NH12-kan*^{r} *leu1 ura4*	2A
NHT1155	*h*$^-$ *klp6(PP1*^{mut}*)-GFP-kan*^{r} *ndc80-NH12-kan*^{r} *leu1 ura4*	2A
NHT293	*h*$^-$ *cut9-665 alp7-myc-kan*^{r} *leu1 ura4*	2B
NHT612	*h*$^-$ *cut9-665 klp5-3FLAG-kan*^{r} *leu1 ura4*	2B
NHT1052	*h*$^-$ *cut9-665 alp7-myc-kan*^{r} *klp5-3FLAG-kan*^{r} *leu1 ura4*	2B
NHT991	*h*$^-$ *klp5-GFP-kan*^{r} *mis6-2mRFP-hph*^{r} *cut12-CFP-nat*^{r} *leu1 ura4*	2C
NHT1068	*h*$^-$ *ndc80-NH12-kan*^{r} *klp5-GFP-kan*^{r} *mis6-2mRFP-hph*^{r} *cut12-CFP-nat*^{r} *leu1 ura4*	2C
NHT238	*h*$^-$ *alp7-GFP-kan*^{r} *leu1 ura4*	3A, 3C, S3A-C
NHT947	*h*$^-$ *alp7-GFP-kan*^{r} *ndc80-NH12-kan*^{r} *leu1 ura4*	3A, 3C, S3C
NHT1048	*h*$^-$ *alp7- 219-268-GFP-kan*^{r} *ndc80-NH12-kan*^{r} *leu1 ura4*	3A
NHT1049	*h*$^-$ *alp7- 269-318-GFP-kan*^{r} *ndc80-NH12-kan*^{r} *leu1 ura4*	3A
NHT1051	*h*$^-$ *alp7- 319-368-GFP-kan*^{r} *ndc80-NH12-kan*^{r} *leu1 ura4*	3A
NHT1057	*h*$^-$ *alp7- 369-429-GFP-kan*^{r} *ndc80-NH12-kan*^{r} *leu1 ura4*	3A, 3C
NHT1053	*h*$^-$ *alp7- 430-474-GFP-kan*^{r} *ndc80-NH12-kan*^{r} *leu1 ura4*	3A
NHT276	*h*$^-$ *alp7::ura4*$^+$ *ndc80-NH12-kan*^{r} *leu1 ura4*	3A
NHT1188	*h*$^-$ *alp7-LA1-GFP-kan*^{r} *ndc80-NH12-kan*^{r} *leu1 ura4*	3C
NHT1189	*h*$^-$ *alp7-LA2-GFP-kan*^{r} *ndc80-NH12-kan*^{r} *leu1 ura4*	3C
NHT944	*h*$^-$ *alp7-LA6-GFP-kan*^{r} *ndc80-NH12-kan*^{r} *leu1 ura4*	3C, S3C

NHT1064	*h*$^-$ *alp7-LA6-myc-kan*^{r} *cen2::hph*^{r}*-lacOp his7*$^+$*::lacI-GFP-ura4*$^+$ *sad1*$^+$*-dsRed-leu2*$^+$ *leu1 ura4*	3D, 5B-F, S4
NHT1011	*h*$^-$ *ndc80-NH12-kan*^{r} *alp7-LA6-myc-kan*^{r} *cen2::hph*^{r}*-lacOp his7*$^+$*::lacI-GFP-ura4*$^+$ *sad1*$^+$*-dsRed-leu2*$^+$ *leu1 ura4*	3D
NHT271	*h*$^-$ *alp7-GFP-kan*^{r} *mis6-2mRFP-hph*^{r} *cut12-CFP-nat*^{r} *leu1 ura4*	4A-B
NHT894	*h*$^-$ *alp7-LA6-GFP-kan*^{r} *mis6-2mRFP-hph*^{r} *cut12-CFP-nat*^{r} *leu1 ura4*	4A-B
NHT294	*h*$^-$ *ndc80-NH12-kan*^{r} *alp7-GFP-kan*^{r} *mis6-2mRFP-hph*^{r} *cut12-CFP-nat*^{r} *leu1 ura4*	4A-B
NHT985	*h*$^-$ *ndc80-NH12-kan*^{r} *alp7-LA6-GFP-kan*^{r} *mis6-2mRFP-hph*^{r} *cut12-CFP-nat*^{r} *leu1 ura4*	4A-B
NHT679	*h- alp7-GFP-kan*^{r} *alp14-mRFP-hph*^{r} *leu1 ura4*	4C
NHT890	*h- alp7-LA6-GFP-kan*^{r} *alp14-mRFP-hph*^{r} *leu1 ura4*	4C
NHT464	*h- mis6-GFP-kan*^{r} *sid4-mRFP-nat*^{r} *aur*^{r}*-mCherry-atb2 leu1 ura4*	4D
NHT954	*h- alp7-LA6-myc-hph*^{r} *mis6-GFP-kan*^{r} *sid4-mRFP-nat*^{r} *aur*^{r}*-mCherry-atb2 leu1 ura4*	4D
NHT911	*h*$^-$ *cut9-665 alp7-myc-hph*^{r} *klp5-3FLAG-kan*^{r} *leu1 ura4*	4E
NHT920	*h*$^-$ *cut9-665 alp7-LA6-myc-hph*^{r} *klp5-3FLAG-kan*^{r} *leu1 ura4*	4E
NHT1325	*h*$^-$ *cut9-665 alp7- 369-429-myc-hph*^{r} *klp5-3FLAG-kan*^{r} *leu1 ura4*	4E
NHT364	*h*$^-$ *ndc80-NH12-kan*^{r} *nuf2-alp7C-kan*^{r} *leu1 ura4*	4F
NHT938	*h*$^-$ *ndc80-NH12-kan*^{r} *nuf2-alp7C-LA6-kan*^{r} *leu1 ura4*	4F

NHT353	*h*$^-$ *nuf2-alp7C-kan*^{r} *leu1 ura4*	4F
NHT915	*h*$^-$ *nuf2-alp7C-LA6-kan*^{r} *leu1 ura4*	4F
NHT875	*h*$^-$ *alp7-LA6-GFP-kan*^{r} *leu1 ura4*	5A, S3B-C
NHT1076	*h*$^-$ *alp7-LA6-GFP-kan*^{r} *mad2::ura4*$^+$ *leu1 ura4*	5A
NHT1418	*h*$^-$ *alp7-LA6-myc-kan*^{r} *mad2::ura4*$^+$ *cen2::hph*^{r}*-lacOp his7*$^+$*::lacI-GFP-ura4*$^+$ *sad1*$^+$*-dsRed-leu2*$^+$ *leu1 ura4*	5B-F
NHT093	*h*$^-$ *ndc80-21-kan*^{r} *leu1 ura4*	S1A
KSH291	*h*$^-$ *ndc80-21-kan*^{r} *mad2::ura4*$^+$ *leu1 ura4*	S1A
NHT1043	*h*$^-$ *sds21::ura4*$^+$ *leu1 ura4*	S1B
NHT1074	*h*$^-$ *ndc80-NH12-kan*^{r} *sds21::ura4*$^+$ *leu1 ura4*	S1B
NHT1401	*h*$^-$ *bub3::ura4*$^+$ *leu1 ura4*	S1C
NHT1405	*h*$^-$ *ndc80-NH12-kan*^{r} *bub3::ura4*$^+$ *leu1 ura4*	S1C
NHT285	*h*$^-$ *ndc80-NH12-kan*^{r} *klp5::ura4*$^+$ *leu1 ura4*	S2B
NHT1000	*h*$^?$ *ndc80-NH12-kan*^{r} *klp5::ura4::klp5(aa.405-883) leu1 ura4 his2/7*	S2B
NHT973	*h*$^-$ *ndc80-NH12-kan*^{r} *klp5C::ura4*$^+$ *leu1 ura4*	S2B
NHT280	*h*$^-$ *ndc80-NH12-kan*^{r} *klp6::ura4*$^+$ *leu1 ura4*	S2B
NHT998	*h*$^?$ *ndc80-NH12-kan*^{r} *klp6::ura4::klp6(aa.411-784) leu1 ura4 his2/7*	S2B
NHT969	*h*$^-$ *ndc80-NH12-kan*^{r} *klp6C::ura4*$^+$ *leu1 ura4*	S2B
NHT536	*h*$^-$ *alp7- 219-268-GFP-kan*^{r} *leu1 ura4*	S3A
NHT428	*h*$^-$ *alp7- 269-318-GFP-kan*^{r} *leu1 ura4*	S3A
NHT538	*h*$^-$ *alp7- 319-368-GFP-kan*^{r} *leu1 ura4*	S3A

NHT539	*h⁻ alp7- 369-429-GFP-kan^r leu1 ura4*	S3A-B
NHT327	*h⁻ alp7- 430-474-GFP-kan^r leu1 ura4*	S3A
NHT239	*h⁻ alp7::ura4$^+$ leu1 ura4*	S3A
NHT804	*h⁻ alp7-LA1-GFP-kan^r leu1 ura4*	S3B
NHT807	*h⁻ alp7-LA2-GFP-kan^r leu1 ura4*	S3B
NHT093	*h⁻ ndc80-21-kan^r leu1 ura4*	S3C
NHT1256	*h⁻ ndc80-21-kan^r alp7-LA6-GFP-kan^r leu1 ura4*	S3C

*Strains were developed for this study except for NHT080, NHT093, NHT238, NHT239, NHT293, NHT327, NHT353, NHT428, NHT536, NHT538, NHT539, NHT1401, NHT1042, NHT1043, KSH291 and wild type 513, which are from our lab stock. *his2=his2-245*; his7=*his7-366*; *leu1=leu1-32*; *ura4=ura4-D18.*

Fig S1. Tang & Toda.

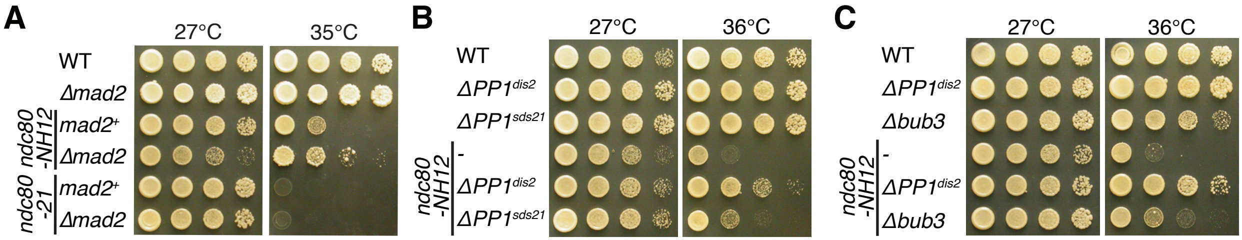

Fig. S1. Temperature sensitivity of the *ndc80-NH12* mutant was suppressed by PP1 deletion, but not Bub3 nor Sds21 deletion.

(**A**) Deletion of *mad2* did not suppress *ndc80-NH12* nor *ndc80-21* mutants. Spot tests were performed on rich agar media and incubated at the temperatures indicated for 3 days.
(**B**) Suppression of *ndc80-NH12* by $PP1^{dis2}$, but not $PP1^{sds21}$, deletion. Spot tests were performed on rich agar media and incubated at the temperatures indicated for 3 days.
(**C**) Modest suppression of *ndc80-NH12* by *bub3* deletion. Spot tests were performed on rich agar media and incubated at the temperatures indicated for 3 days.

Fig S2. Tang & Toda.

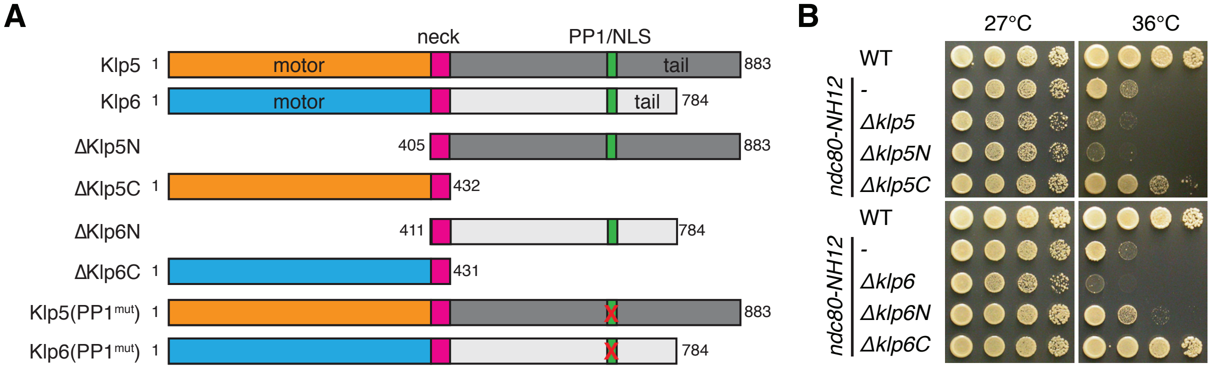

Fig. S2. Temperature sensitivity of the *ndc80-NH12* mutant was not suppressed by Klp5/Klp6 deletion.

(**A**) Schematic presentation of Klp5 and Klp6 kinesin 8 molecules. The *klp5(PP1^{mut})* mutant contains four amino acid replaced to alanine at the position V683, F685, V694 and F696; whereas two mutations V674A and F676A were introduced into the *klp6(PP1^{mut})* mutant (Meadows et al., 2011).
(**B**) Individual strains were constructed and spot tests were performed. Plates were incubated for 3 days under the indicated conditions.

Fig S3. Tang & Toda.

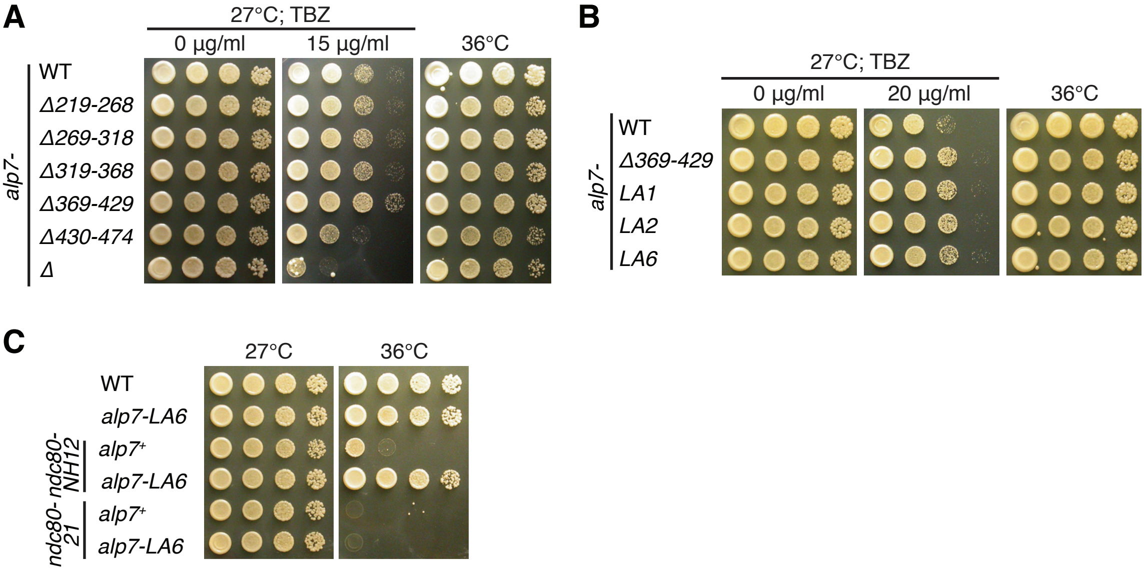

Fig. S3. Introduction of *alp7-LA6* rescues the *ndc80-NH12*, but not *ndc80-21* phenotypes.

(**A**) Deletion of amino acids 369-429 of Alp7 results in modest TBZ resistance. Spot tests were performed on rich agar media and incubated at the temperatures indicated for 3 days.
(**B**) Alp7-LA1, Alp7-LA2 and Alp7-LA6 showed modest TBZ resistance as in Alp7-Δ369-429. Spot tests were performed on rich agar media and incubated at the temperatures indicated for 3 days.
(**C**) Introduction of *alp7-LA6* rescues *ndc80-NH12*, but not *ndc80-21*. Spot tests were performed on rich agar media and incubated at the temperatures indicated for 3 days.

Fig S4. Tang & Toda.

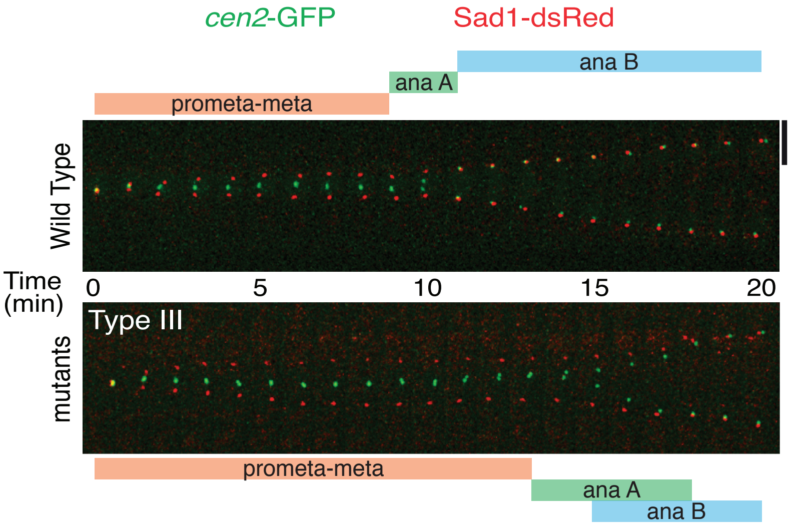

Fig. S4. The *alp7-LA6* mutant showed slow anaphase A centromere movement.

Visualisation of *cen2* movement in *alp7-LA6* mutant. Scale bar, 5 µm. Complete mitotic progression is shown for representative images as shown in Figure 5E. See movie S4 (Type III).

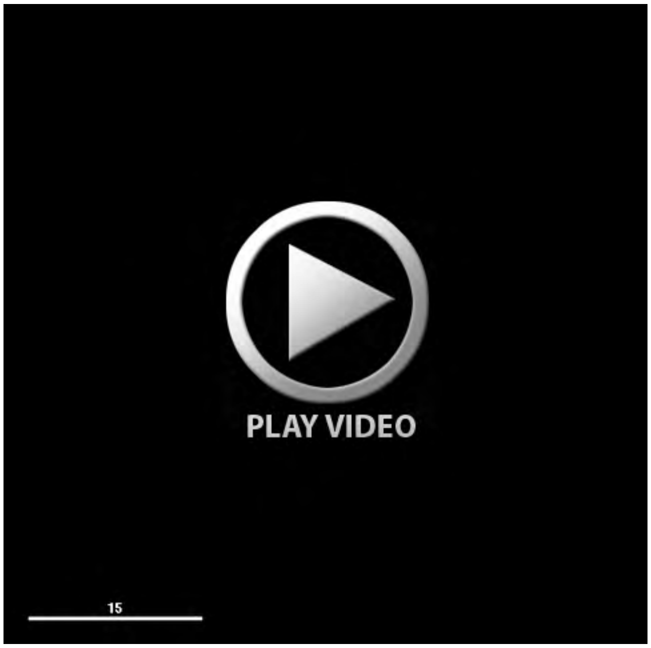

Movie S1: *cen2*-GFP movement during mitosis in wild type (corresponding to Fig.1C, top).
Centromeres on chromosome II are visualised with GFP (*cen2*-GFP) in a cell carrying a SPB marker (Sad1-dsRed).

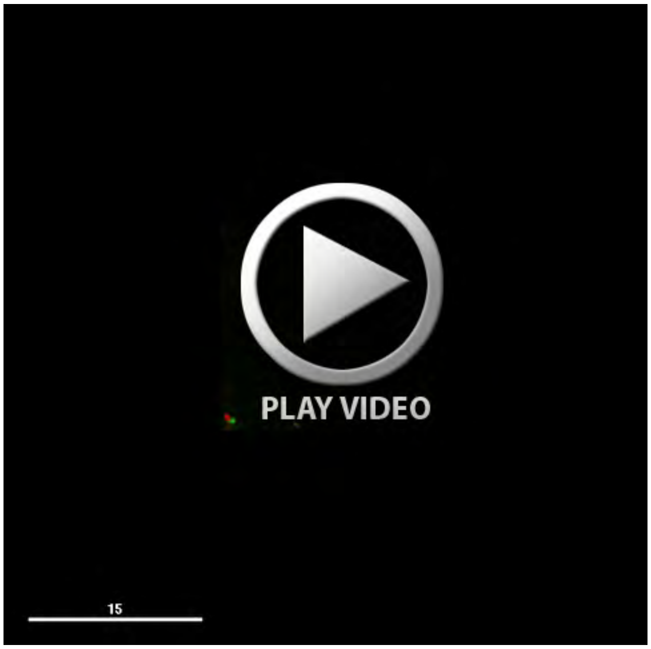

Movie S2: *cen2*-GFP movement during mitosis in *ndc80-NH12* (corresponding to Fig. 1C, Type I).
Centromeres on chromosome II are visualised with GFP (*cen2*-GFP) in a cell carrying a SPB marker (Sad1-dsRed). Note that *cen2*-GFP does not split, but moves back and forth between the two poles for > 20 min.

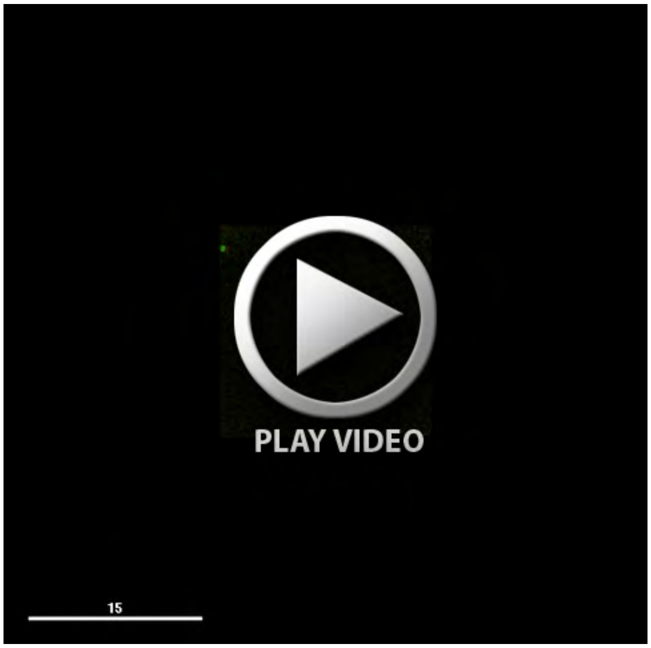

Movie S3: *cen2*-GFP movement during mitosis in *ndc80-NH12* (corresponding to Fig. 1C, Type II).
Centromeres on chromosome II are visualised with GFP (*cen2*-GFP) in a cell carrying a SPB marker (Sad1-dsRed). Note that *cen2*-GFP signals split into two, but move to one pole but not the other, causing mis-segregation of *cen2*.

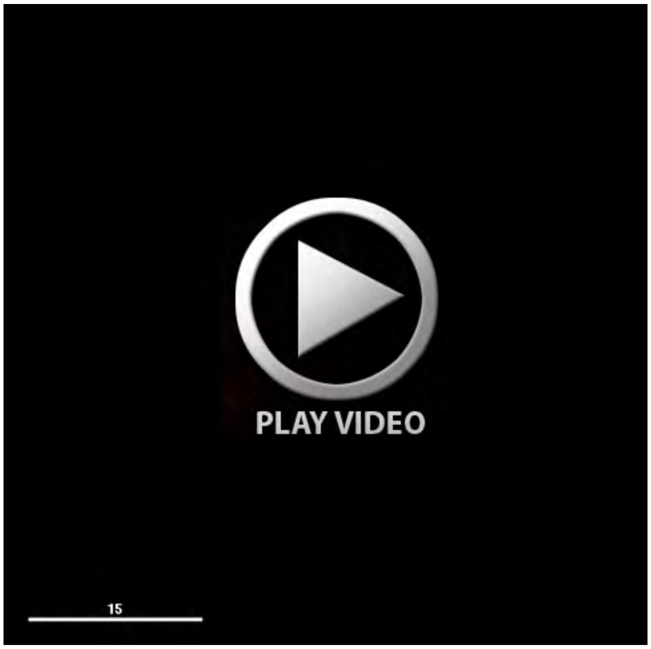

Movie S4: *cen2*-GFP movement during mitosis in *alp7-LA6* (corresponding to Fig. 5E and supplementary material Fig. S4, Type III).
Centromeres on chromosome II are visualised with GFP (*cen2*-GFP) in a cell carrying a SPB marker (Sad1-dsRed). Note that *cen2* movement during anaphase A is slowed down and anaphase B takes place before completion of anaphase A.
